# EARLY CLINICAL UTILITY DATA OF A MULTI-ANALYTE BLOOD BIOMARKER TEST FOR EVALUATING COGNITIVE IMPAIRMENT

**DOI:** 10.1093/geroni/igae098.2972

**Published:** 2024-12-31

**Authors:** Mark Monane, David Merrill, Kim Johnson, Darren Gitelman, Demetrius Maraganore, Leslie Jacobs, Yan Jiang, Joel Braunstein

**Affiliations:** C2N Diagnostics, St. Louis, Missouri, United States; Pacific Brain Health Center, Santa Monica, California, United States; Duke University, Durham, North Carolina, United States; Advocate Lutheran General Hospital, Park Ridge, Illinois, United States; Tulane University School of Medicine, New Orleans, Louisiana, United States; C2N Diagnostics, St. Louis, Missouri, United States; Stat4ward, Pittsburgh, Pennsylvania, United States; C2N Diagnostics, St. Louis, Missouri, United States

## Abstract

There is an important unmet need for timely, noninvasive, low-burden diagnostic tools to aid in Alzheimer’s disease evaluation. The AAA-BBM2 (PrecivityAD2™) blood test combines plasma amyloid beta (AB)42/40 Ratio and phosphorylated-tau217/non-phosphorylated-tau217 Ratio (%p-tau217) into a clinically-validated algorithm. The test result, the Amyloid Probability Score 2 (APS2), determines the likelihood of brain amyloid, the pathological hallmark of Alzheimer’s disease, on amyloid PET scan: the test result has previously demonstrated 88% sensitivity and 89% specificity. The objective of the QUIP II Study (NCT06025877) is to evaluate the clinical utility of this blood biomarker test with APS2 result among cognitively symptomatic patients presenting to memory specialists. To date, 52 symptomatic patients (median age 75, 65% female, 77% white) from 7 memory centers received the AAA-BBM2 test. Concordance with intended use of the test was 100% (52/52). Positive APS2 results were noted in 26 patients (50%). From pre- to post-test, clinician-reported probability of AD decreased from 48% to 16% among negative APS2 patients (p< 0.0001), and increased from 71% to 93% among positive APS2 patients (p< 0.0001). The composite endpoint of change in AD diagnostic certainty, drug therapy, or brain amyloid evaluation pre- and post-AAA-BBM2 testing was 79% (p < 0.0001 versus a pre-specified threshold of 20% clinically meaningful change). We believe that the AAA-BBM2 test showed clinical utility in its association with changes in clinicians’ diagnostic certainty and clinical management among patients presenting with cognitive impairment. This blood biomarker test may allow memory specialists to guide patients to anti-AD therapies and facilitate other diagnostic considerations.

